# Transport and inhibition mechanisms of human creatine transporter

**DOI:** 10.1038/s41421-025-00801-4

**Published:** 2025-05-05

**Authors:** Jiahui Chen, Yimin Zhang, Nanhao Chen, Jingpeng Ge, Jie Yu

**Affiliations:** 1 Interdisciplinary Research Center on Biology and Chemistry, Shanghai Institute of Organic Chemistry, Chinese Academy of Sciences, Shanghai, China; 2 University of Chinese Academy of Sciences, Beijing, China; 3 Center for Quantitative Biology, Academy for Advanced Interdisciplinary Studies, Peking University, Beijing, China; 4 School of Life Science and Technology, ShanghaiTech University, Shanghai, China; 5 Shanghai Key Laboratory of Aging Studies, Shanghai, China

**Keywords:** Cryoelectron microscopy, Mechanisms of disease

Dear Editor,

Creatine, a guanidine-based molecule, acts as a rapid phosphate energy buffer in its phosphorylated form by donating a phosphate group to ADP, thereby regenerating ATP^1^. This process is crucial for maintaining ATP levels and energy metabolism in tissues with high and variable energy requirements, such as the brain and muscles^2^. Additionally, creatine functions as a compatible osmolyte in the brain and a neuromodulator or neurotransmitter by interacting with *N*-methyl-d-aspartate receptors and GABA_A_ receptors^3–5^. Creatine is obtained by the diet or synthesized by endogenous enzymes, and specifically transported into the cell using the creatine transporter (CRT)^6^, a sodium- and chloride-dependent transporter, belonging to the solute carrier 6 (SLC6) family. Impaired function of CRT leads to intracellular creatine deficiency^7^, resulting in X-linked creatine transporter deficiency (CTD), a disorder characterized by intellectual disability, speech and language delay, behavioral abnormalities, and seizures. Over 80 CRT pathogenic variants have been identified, but no effective treatment is currently available for this condition. Determining the atomic structure of CRT would greatly aid in developing therapeutic strategies targeting these pathogenic variants.

Conversely, overexpression of CRT has been identified in several types of cancers. As the growth and progression of the tumor under hypoxic conditions require ATP and phosphocreatine, inhibiting intracellular creatine levels leads to reduced ATP levels, thereby suppressing cancer cell development. RGX-202 (Ompenaclid, RGX), a compound in phase II clinical trials, has been shown to competitively inhibit creatine transport by CRT, efficiently inducing tumor apoptosis and suppressing colorectal cancer growth^8^. Meanwhile, RGX has also been proven to be an alternative substrate of CRT^9^. However, its binding mode and mechanism of inhibition remain to be elucidated.

To gain insights into the substrate recognition and inhibition mechanism, we expressed the full-length wild-type (WT) human CRT in HEK293 cells and performed [^14^C] creatine uptake assay (Fig. 1a). The results showed that CRT displayed a Michaelis constat (*K*_m_) value of 106.3 μM, indicating that HEK293 cells express functional CRT. To facilitate structural analysis of small membrane proteins, we purified CRT in the presence of LMNG, which exhibits a decent biochemical behavior on size-exclusion chromatography and thin micelles in the 2D class average (Supplementary Fig. S1). We determined the cryo-EM structures of CRT in the apo (CRT-apo), and in the presence of substrate creatine (CRT–creatine) or inhibitor RGX (CRT–RGX), at the resolutions of 3.29 Å, 3.40 Å, and 3.39 Å, respectively (Supplementary Figs. S1–S3 and Table S1). Details for sample preparations, data acquisition, and analysis are summarized in the supplementary information.

**Fig. 1:**
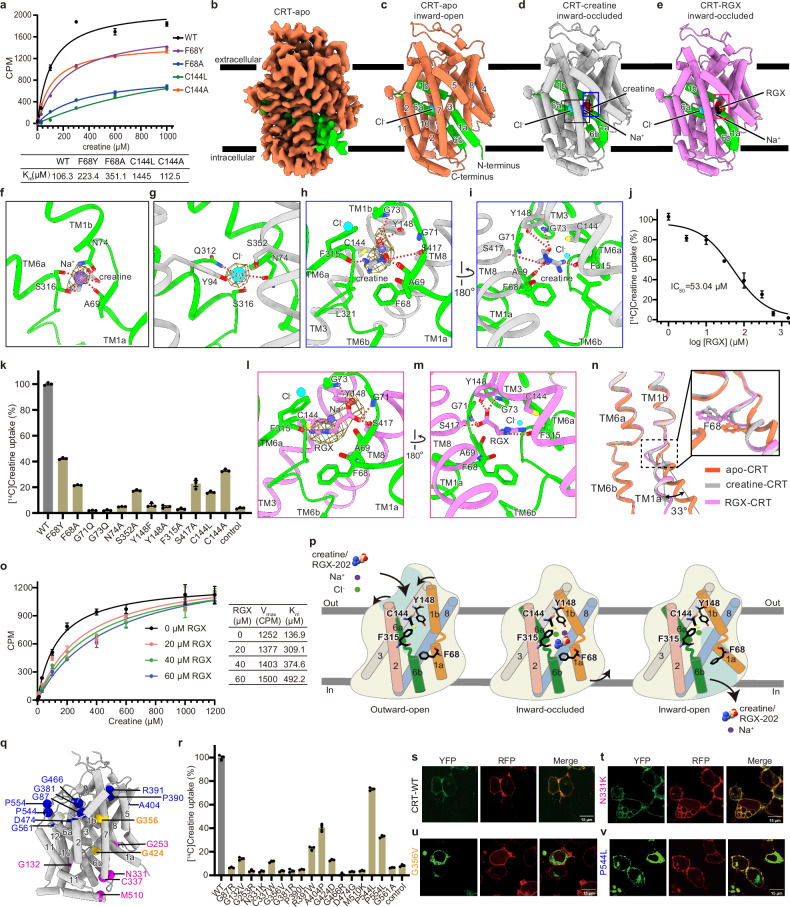
Molecular basis for creatine and RGX recognition by human CRT. **a** Plots of [^14^C]creatine saturation binding to WT and the indicated mutants of CRT. The corresponding *K*_m_ values are indicated in the table below. Symbols show the mean of three independent replicates, with error bars representing the SEM. **b** Cryo-EM construction map of CRT in the apo form. **c**–**e** Coordinates of CRT in the apo (**c**), in complexes with creatine (**d**), and inhibitor RGX (**e**), viewed parallel to the membrane. CRT-apo is the inward-open conformation, and both CRT–creatine and CRT–RGX are in the inward-occluded conformation. Two gating helices TM1 and TM6 are highlighted in green. **f**, **g** The coordination of Na^+^ (**f**) and Cl^–^ (**g**) ions in the central binding site of CRT–creatine. The contour level is 0.23. **h**, **i** Different views of the interactions of creatine in the central binding site of CRT–creatine. The contour level is 0.23. **j** Concentration-inhibition curve of [^14^C]creatine uptake by RGX in the CRT. **k** Measuring the comparative transport activities of WT CRT and the indicated CRT variants using [^14^C]creatine uptake. The activities of CRT variants are normalized to that of WT CRT. Data are means with SEM of three independent experiments. **l**, **m** Different views of interactions of RGX in the central binding site of CRT–creatine. The contour level is 0.23. **n** Superimposition of the structures of the CRT-apo, CRT–creatine and CRT–RGX to illustrate the conformational changes in the TM1a. The positions of ions, creatine, and RGX are marked with rectangles. **o** [^14^C]Creatine saturation binding experiments of CRT in different concentrations of RGX, with corresponding V_max_ and *K*_m_ values indicated in the right table. RGX was mixed with [^14^C]creatine directly, without preincubation with cells. Data are represented with the SEM of three independent experiments. **p** Cartoons depict the mechanism of transport and inhibitor action. Structural elements and critical residues for substrate binding and gating are shown. TM1 and TM6 form the substrate translocation pathway. RGX, working as the competitive inhibitor of CRT, directly competes with creatine for binding to the substrate site and transportation. **q** Distribution of 17 CTD disease-related mutations in the CRT. The mutations in the extracellular side, the middle of TM, and the intracellular side are labeled with blue, gold, and magenta balls, respectively. **r** Evaluating the comparative transport activities of WT CRT and its 17 CTD disease-related variants using [^14^C]creatine uptake. The activities of CRT variants are normalized to that of WT CRT. Data are means with SEM of three independent experiments. **s–v** Subcellular localization of selected CTD-related CRT variants. WT CRT and its variants are labeled in green, and neuromodulin (plasma membrane marker) was labeled in red. N331K (**t**), G356V (**u**), and P544L (**v**) are located on the intracellular side, TM helix, and extracellular side of CRT, respectively. Fluorescence images for the remaining CTD-related CRT variants are provided in Supplementary Fig. S6.

The quality of cryo-EM maps enables us to build most regions of CRT, while the unstructured N-terminus (residues 1–55), extracellular loop (residues 188–201), and C-terminus (residues 604–635) are unsolved, likely due to their intrinsic flexibility (Fig. 1b, c). The structure of CRT adopts a typical LeuT fold, consisting of 12 transmembrane (TM) helices, with two gating helices, TM1 and TM6, each divided into segments TM1a/1b and TM6a/6b. In the structure of CRT-apo, TM1 and TM6 form a translocation pathway accessible exclusively from the cytoplasmic side, suggesting that CRT-apo is in the inward-open state (Supplementary Fig. S8e). Notably, in the CRT-apo, a distinct density has been identified as a putative Cl^–^ ion, with no additional densities observed for Na^+^ ions (Fig. 1c; Supplementary Fig. S8f). CRT–creatine is in an inward-occluded conformation (Supplementary Fig. S8e), characterized by the occlusion of the intracellular pathway by TM1a (Fig. 1d), similar to the substrate-bound structures of GAT1^10^ and GlyT1^11^ (Supplementary Fig. S8a–d). In the structure of CRT–creatine, Na^+^1, Cl^–^, and creatine were identified in the central binding site, while no discernible density was observed for the second Na^+^2 ion, likely due to relatively poor density in the Na^+^2 binding site (Fig. 1f, g). Na^+^1 and Cl^–^ are occupied in the conserved binding sites, as observed in the structures of other Neurotransmitter Sodium Symporter (NSS) family members, such as GAT1 and GlyT1^10,11^. The Na^+^1 ion is likely chelated by five coordination with surrounding residues, including the side chain and carbonyl oxygen of Ser316, the amino group and carbonyl oxygen of Asn74, and the backbone of Ala69 (Fig. 1f). Additionally, Na^+^1 is further stabilized by interactions with hydroxyl group and carbonyl oxygen of creatine, with the distances of 3.2 Å and 3.6 Å, respectively. Consistent with structural observation, substitution of Asn74 with Ala abolishes transport activity (Fig. 1k; Supplementary Fig. S5r). Cl^–^ ion forms possible pentagonal coordination bonds with side chains of Asn74, Tyr94, Gln312, Ser316, and Ser352 (Fig. 1g). Similarly, mutation of Ser352 to Ala leads to a nearly complete loss of CRT function (Fig. 1k; Supplementary Fig. S5r). These findings support the essential roles of Na^+^ and Cl^–^ ions in CRT function.

In the central binding site of CRT–creatine, creatine is stabilized by potential hydrogen bonds with the side chain of Tyr148, the hydroxyl group of Ser417, the carbonyl oxygens of Ala69 and Phe315, and the nitrogen atom of backbone Gly73 (Fig. 1h, i). The Ser417Ala mutant causes a dramatic reduction in activity (Fig. 1k; Supplementary Fig. S5r). Three aromatic residues Phe68, Tyr148, and Phe315 surround and stabilize the guanidine group of creatine through π–π interactions. Substitution of Tyr148 by Phe or Ala results in the abolishment of transporter function (Fig. 1k; Supplementary Fig. S5r), suggesting that both the aromatic ring and hydroxyl group of Tyr148 are critical in substrate binding. Mutating Phe315 to Ala completely disrupts the transport activity of CRT (Fig. 1k). The importance of Phe315 has been discovered in previous studies, where its deletion was linked to CTD, a condition characterized by intellectual disability, epilepsy, and speech delay^12^. Cys144, proximity to the guanidine group of creatine, is unique to CRT and corresponds to more hydrophobic residues in other members of SLC6 family (Supplementary Fig. S4). Studies have shown that deprotonated Cys144 forms a salt bridge with guanidine group of creatine, and mutations on Cys144 affect transport activity and exhibit a 10-fold increase in GABA transport^13^. Consistent with these findings, replacing Cys144 with either the hydrophobic residue Leu or the nonpolar residue Ala significantly reduces the activity of CRT and increases *K*_m_ for creatine (Fig. 1a, k).

To explore the molecular basis for distinct substrate specificities among the members in the NSS family, we superimposed the binding pockets of CRT–creatine with those of GAT1–GABA^10^, GlyT1–glycine^11^ and DAT–dopamine^14^, respectively. Structural comparisons reveal that while CRT and GAT1 share a similar substrate-binding pose, key non-conserved residues, such as Cys144 and Phe68 in CRT, contribute to substrate specificity. By contrast, GlyT1 accommodates glycine in a compact pocket shaped by W322, and DAT exhibits a distinct binding mode where Asp79 recognizes dopamine’s amine group, a position occupied by Gly71 in CRT. These differences highlight the critical role of non-conserved residues in defining substrate specificity among NSS transporters (Supplementary Fig. S8a–d).

RGX is a potent competitive inhibitor of CRT and has been shown to significantly suppress tumor growth in clinical trials^8^. RGX inhibits the creatine transport activity with an IC_50_ value of 53.04 µM (Fig. 1j). To verify the competitive inhibition of RGX, we applied RGX simultaneously with the substrate in the [^14^C] creatine uptake assay. At saturating concentration, the maximal reaction velocity (V_max_) remains largely unchanged, but the *K*_m_ is substantially increased (Fig. 1o), confirming RGX acts as a competitive inhibitor of CRT. To investigate the inhibition mechanism by RGX, we determined the structure of CRT–RGX in an inward-occluded state (Supplementary Fig. S8e). The structure of CRT–RGX aligns closely with CRT–creatine, with a root mean square deviation value of 0.45 Å. TM1a is in a similar configuration, with one Na^+^ and one Cl^–^ ion occupying the same position (Fig. 1e). RGX adopts the same binding pose as creatine, with its carboxylate group orient toward the unwound region of TM1 and its guanidine group is nestled among three aromatic residues Phe68, Tyr148 and Phe315 (Fig. 1l, m). The residues such as Ala69, Gly73, Tyr148, Phe315, and Ser417, which interact with creatine, also contribute to stabilizing RGX through hydrogen bonds. Compared to creatine, RGX is larger, featuring an additional alkyl group. Its carboxylate group extends closer to the unwound region of TM1, forming two additional potential hydrogen bonds with backbone nitrogen atoms of Gly71 and Leu72 (Fig. 1l, m).

Additionally, we performed molecular dynamics (MD) simulations to investigate the stability of creatine and RGX in the structure of CRT. The simulation results reveal that the creatine binds to CRT in two distinct modes. In the first mode, creatine coordinates with Na^+^ ion (Supplementary Fig. S7a–c) while maintaining the hydrogen bond interaction with Tyr148, Gly73, and Ser316, consistent with the binding mode observed in our structure (Supplementary Fig. S7b). In the second mode, the carboxylate group of creatine no longer coordinates with Na^+^, but instead forms additional interactions with surrounding residues, such as Leu72 and Leu413 (Supplementary Fig. S7c). By contrast, RGX does not coordinate with the Na^+^ ion and adopts a single binding mode after 300 ns MD simulations, resembling the second binding mode of creatine (Supplementary Fig. S7d–f). Based on these findings, we hypothesize that the larger size of RGX contributes to its stability in the central binding pocket.

Comparison of the inward-open (CRT-apo) and inward-occluded (CRT–creatine and CRT–RGX) structures reveals that the most evident conformational changes are located in TM1a (Fig. 1n). In the structure of CRT-apo, TM1a bends ~33° toward the membrane plane, thereby opening the intracellular translocation pathway. Phe68 in the TM1a, sitting in the entrance of the intracellular gate, interacts and stabilizes the binding of substrate, and sterically blocks the release of creatine from the central binding pocket (Fig. 1n), likely playing important roles in the substrate recognition and gating of CRT. Supporting this observation, replacing Phe68 either with Tyr or Ala substantially reduced transport activity and markedly increased *K*_m_ values (Fig. 1a, k). The conformational changes primarily involved the displacement of TM1a, a phenomenon also observed in GAT1 and GlyT1 during the transition from inward-occluded to inward-open state^10,11^.

Based on the inward-open and inward-occluded structures determined in this study, along with previously solved outward-open structures of the members in the SLC6 family, we propose that creatine binds to the central binding site in the outward-open conformation, undergoes a conformational transition driven by the presence of Na^+^ and Cl^–^ ions, becomes exposed to the cytoplasmic side in the inward-occluded state, and is subsequently released to the cytoplasm in the inward-open conformation along with Na^+^ ion. Meanwhile, the Cl^−^ ion remains in its conserved position and is not released. (Fig. 1p). RGX inhibits CRT by directly competing with creatine for binding to the substrate site and transport (Fig. 1p).

The structure of human CRT provides an atomic molecule model to pinpoint mutations associated with X-linked CTD. We mapped 17 clinically relevant CRT variants and found that the mutations are scattered throughout the transporter, including TMs, extracellular loops, and intracellular loops (Fig. 1q). Previous studies suggested that these mutations impair transport activity because of protein misfolding and incorrect cellular localization^15^. Of the 17 disease-related variants, 16 exhibit proper behavior, while one variant fails to express, as indicated by fluorescence-detection size exclusion chromatography results (Supplementary Fig. S5a–q). Our [^14^C] creatine transport assay revealed that most of the 16 mutants lack activity, although a few, such as A404P, P544L and P554L, retain partial activity (Fig. 1r). Moreover, the confocal microscopy imaging shows that the mutations on the intracellular side are correctly localized to the plasma membrane (Fig. 1s, t; Supplementary Fig. S6). By contrast, mutations on the extracellular side and TM are either exclusively retained within in the cell or partially localized to both the plasma membrane and intracellular compartments (Fig. 1u, v; Supplementary Fig. S6). These findings suggest that intracellular mutations likely affect transport activity through a mechanism unrelated to incorrect cellular localization, potentially influencing the gating or stability of the transporter.

In this study, we elucidated the structures of CRT in the apo, in complexes with substrate creatine, and with a clinical trial competitive inhibitor RGX. These structures reveal the binding sites for ions, substrate, and inhibitor as well as conformational transition from the occluded to inward-open states. In combination with functional assays, we confirmed the competitive action and inhibition mechanism of RGX and identified the molecular determinants of CRT’s substrate specificity and gating mechanisms. We further investigated how pathogenic variants impact CRT function and cellular localization through fluorescence microscopy. Overall, this study provides an insight into the mechanisms underlying substrate recognition and competitive inhibition in CRT, and advances the understanding of how CTD-related variants impact CRT function and cellular localization, thereby paving the way for future drug discovery targeting CRT.

## Supplementary information

Supplementary figures and tables

## Data Availability

The 3D cryo-EM density maps and coordinates of CRT-apo, CRT–creatine, and CRT–RGX have been deposited in the Electron Microscopy Data Bank (EMDB) under the accession numbers EMD-62521, EMD-62527 and EMD-62528 and the Protein Data Bank (PDB) under accession codes 9KR7, 9KRH and 9KRI, respectively.
