# Supplementary material for: Transport and inhibition mechanisms of human creatine transporter
Source: Cell Discov. 2025 May 5;11:43. doi: 10.1038/s41421-025-00801-4 (PMC12053652; doi:10.1038/s41421-025-00801-4)
Supplement: Supplementary file 1 — Supplementary figures and tables [file 41421_2025_801_MOESM1_ESM.pdf]

Supplementary Information for

Transport and inhibition mechanisms of human creatine transporter

Jiahui Chen[1, 2#], Yimin Zhang[1, 2#], Nanhao Chen[3#], Jingpeng Ge[4*], Jie Yu[1, 5*]

[1]Interdisciplinary Research Center on Biology and Chemistry, Shanghai Institute of Organic Chemistry, Chinese Academy of Sciences; Shanghai, 201210, China.

[2]University of Chinese Academy of Sciences; Beijing, 100049, China.

[3]Center for Quantitative Biology, Academy for Advanced Interdisciplinary Studies, Peking University; Beijing, 100871, China.

[4]School of Life Science and Technology, ShanghaiTech University; Shanghai, 201210, China.

[5]Shanghai Key Laboratory of Aging Studies, Shanghai

#These authors contributed equally to this work.

*Corresponding author Email: yujie@sioc.ac.cn.

The PDF file includes:

Materials and Methods

Supplementary References

Supplementary Figs. S1-S8

Supplementary Table S1

Materials and Methods

Clone, expression and purification of human creatine transporter (CRT)

The coding sequences for full-length human CRT (*SLC6A8*, UniProt ID: P48029) were cloned into the pEGBacMam vector with a C-terminal tag containing a 3C protease cleavage site, a Yellow Fluorescent Protein (YFP) and a Twin-Strep affinity tag. CRT was expressed in HEK293S GnTI^{-} cells cultured in Freestyle 293 Expression Medium (Gibco) supplemented with fetal bovine serum (FBS) (1% v/v). When the cell density reached approximately 3.0×10^6 cells/ml, one liter of cells was transiently transfected with the 1 mg of CRT plasmids, which were pre-mixed with polyethylenimine (PEI, Yeasen Biotechnology) at a 1:4 (w/w) ratio. Cells were then maintained at 37 °C with 5% CO_2 for 10 hours. Subsequently, 10 mM sodium butyrate was added to boost protein expression, and the cells were shifted to 30 °C for an additional 48 hours before being collected.

All purification steps were performed at 4 °C or on ice unless otherwise specified. The cell pellets were resuspended with the buffer containing 20 mM Tris pH 8.0, 150 mM NaCl, 1% (w/v) n-dodecyl-β-D-maltopyranoside (DDM, Anatrace), 0.2% (w/v) cholesteryl hemisuccinate (CHS, Anatrace), 0.8 μM aprotinin, 2 μg/ml leupeptin, 2 mM pepstatin A and 1 mM phenylmethylsulfonyl fluoride, and incubated for 2 hours. Insoluble debris was removed by centrifugation at 186,009g for 1 h. The supernatant was passed through a 0.45-μm polystyrene membrane and incubated with the 4 ml Strep-Tactin resin for 1h. The resin was pre-equilibrated with the buffer A, consisting of 20 mM Tris pH 8.0, 150 mM NaCl, 0.01% (w/v) lauryl maltose neopentyl glycol (LMNG, Anatrace), 2 mM ATP and 2 mM $MgCl_2$. Then the resin was washed with 30 column volumes of buffer A. The protein was eluted by buffer A supplemented with 5 mM desthiobiotin. The elution was digested by 3C protease for 1h and further purified by size-exclusion chromatography on a Superose 6 10/300 GL column (GE Healthcare) in the presence of buffer A. The protein peak fractions were inspected by SDS-PAGE and collected for cryo-EM analysis.

Cryo-EM sample preparation and data acquisition

The final concentration of protein samples for cryo-EM sample preparation was approximately 1.5 mg/mL. For the CRT-creatine or RGX-202 complexes, creatine (Sigma-Aldrich) and RGX-202 (Sigma-Aldrich) were added to the sample at final concentrations of 2 mM and 5 mM, respectively. An aliquot of 2.5 μL of the sample was applied to glow-discharged Quantifoil R2/1 200-mesh gold holey carbon grids and incubated for 10 s. The grids were then blotted for 3 s under 95% humidity at 8 ℃, followed by plunge freezing into liquid ethane cooled by liquid nitrogen, performed in the Vitrobot Mark IV. Cryo-EM grids were imaged on a Titan Krios microscope (Thermo Fisher Scientific) operating at 300 kV, equipped with a K3 direct electron detector (Gatan) and an energy filter for data collection. Movies were captured using EPU software at a calibrated magnification of 81,000× in the super-resolution mode, resulting in 0.5275 Å per pixel. Data were collected with a defocus range of -1.0 to -2.0 μm. Each micrograph was recorded with a total dose of 50 e-/Å^2 at a dose rate of ~ 25 e-/pixel/s.

Cryo-EM data processing

All the data processing procedures were conducted in CryoSparc v4.5.3[1]. In total, 5,622, 12,818 and 8,222 cryo-EM movies were collected for CRT-apo, CRT-creatine and CRT-RGX, respectively.

For the CRT-apo dataset, the image stacks were motion corrected using Patch Motion Correction, followed by contrast transfer function (CTF) estimation using Patch CTF. Micrographs with a CTF fitting resolution worse than 5 Å were removed, resulting in 5,272 micrographs. Blob picker was used to pick particles (diameter 80-120 Å), followed by several rounds of 2D classification to generate good 2D classes as the template for template picking. Particles picked by the blob picker and template picker were combined, and duplicate entries (within ~30 Å) were removed. As a result, 7,221,317 particles were subjected to several rounds of 2D classification. The 2D classes displaying clear secondary structure features were used for ab-initio reconstructions and heterogeneous refinements. Iterative rounds of heterogenous refinement for multi-reference 3D classification were performed using one good

reference and five biased maps to exclude undesirable particles, resulted in a best class containing 456,700 particles. To further remove junk particles, several rounds of 2D classifications and heterogenous refinements were conducted, giving rise to a subset of 261,670 particles for non-uniform refinement. Local refinement was performed, using a custom mask, produced a 3.29 Å resolution map according to gold-standard Fourier shell correlation (GSFSC) criterion. Similar procedures were applied to the CRT-creatine and CRT-RGX datasets, yielding final subsets of 200,112 and 262,011 particles, respectively, which produced reconstruction maps at the resolution of 3.34 Å and 3.39 Å based on GSFSC criterions.

Model building

The initial atomic model of CRT was generated by AlphaFold2 and fitted into the 3D reconstruction map of CRT-apo by UCSF ChimeraX[2]. The Cα backbone and side chains of residues were manually adjusted and rebuilt in Coot[3], followed by the refinement in Phenix. The resulting CRT-apo model served as the initial template for the structures of CRT-creatine and CRT-RGX. Geometry constraints for creatine and RGX was produced by elBow tool in Phenix[4]. The final models for all three datasets were refined against the corresponding maps using phenix.real_space_refine module in Phenix with secondary structure and geometry restraints applied. The model validation was performed using MolProbity[5] and Phenix[4], with model refinement and validation statistics summarized in the Extended Table 1. All figures were prepared using UCSF ChimeraX[2].

[^{14}C]Creatine transport assay

For the transport assay, constructs of wild-type and mutant CRT without C-terminal tag were used. HEK 293-T cells were first cultured in 24-well plate coated with Poly-L-Lysine (Sigma-Aldrich) and transfected with the plasmid and polyJeT (SignaGen) when density reached at ~70-80%. Cells without transfection were used as blank control. The cells were cultured at 37 °C with 5% CO_2 for 36 hours. After removing the growth medium, the cells were washed twice with 500 μL pre-warmed KRH buffer (120 mM NaCl, 4.7 mM KCl, 1.2 mM $MgSO_4$, 2.2 mM $CaCl_2$, 1.2 mM

KH_2PO_4, 10 mM glucose, 25 mM HEPES pH 7.4). The cells were then incubated with 200 μL of KRH buffer containing 200 μM cold creatine and 0.2 μM [^{14}C]creatine (American Radiolabeled Chemicals, ARC-0176, 55 mCi/mmol) for 45 min at 37 °C. The molar ratio of [^{14}C]creatine and cold creatine was maintained at 1:1000 for each reaction. For the time-course experiment, the uptake was terminated at different time (5min, 10min, 20min, 30 min, 45 min, 60 min, 80 min, 90 min and 100 min). For the saturation uptake kinetic assay, uptake was initiated by adding a series of concentrations of creatine (0.3 μM, 1 μM, 3 μM, 10 μM, 30 μM, 100 μM, 300 μM, 600 μM and 1000 μM). Following incubation, the uptake medium was removed, the cells were washed twice with 500 μL of ice-cold KRH buffer. The cells were solubilized in KRH buffer containing 1% Triton X-100, and aliquots were taken for scintillation counting.

For the uptake inhibition assay, cells were preincubated with 100 μL different concentrations of RGX (1-1500 μM) in KRH buffer (2×) for 3 min at 37 °C. The uptake was initiated by adding equal volumes of KRH buffer (2×) containing a mixture of 0.4 μM [^{14}C]creatine and 0.4 mM cold creatine, and were stopped by adding ice-cold KRH buffer. The cells were then washed and lysed as described above. To assess the competitive inhibition effect, RGX (20, 40 or 60 μM) was added to the KRH buffer containing varying concentrations of creatine directly in a 96-well plate, without preincubation with cells. After incubation for 45 min, the cells were washed twice with 100 μL of ice-cold KRH buffer. Cells were lysed in 50 μL 1% Triton X-100, and the lysates were transferred to IsoPlate-96 plates (PerkinElmer). The radioactivity content was measured by liquid scintillation counting.

For calibrating the expression of CRT variants, constructs of wild-type and mutant CRT with a C-terminal YFP tag were employed. The YFP fluorescence intensity of each construct was measured using fluorescence-detection size exclusion chromatography (FSEC)[6]. The fluorescence intensity ratios of wild-type to mutants were applied to normalize the uptake values for each mutant. Samples were also taken to determine protein concentration using a BCA protein assay kit (ThermoFisher). All assays were carried out in triplicate, and statistical analyses were performed using

GraphPad Prism 9.

Fluorescence microscopy

To assess whether creatine transporter deficiency (CTD) variants impact CRT function by altering cellular targeting, we co-expressed YFP-tagged wild-type CRT or its variants with RFP-tagged neuromodulin, a plasma membrane marker, in HEK 293T cells. One day prior to transfection, cells were seeded onto 35 mm glass bottom dishes (Cellvis). The cells were transfected with the plasmid and polyJeT transfection reagent when density reached at approximately 50%, followed by incubation at 37 °C with 5% CO_2 for another 24 h. Live cells were visualized using a confocal laser scanning microscope with a ×60 magnification under immersion oil. Image analysis was conducted using ImageJ software. Colocalization of wild-type CRT or its variants with plasma membrane was quantified by Pearson's correlation coefficient. Values of 1 and -1 represent 100% co-localization and an inverse distribution, respectively.

Cell surface biotinylation and immunoblot analysis

Constructs of wild-type and mutant CRT with a C-terminal flag tag were used. HEK293F cells were cultured transiently transfected with plasmids of CRT WT and mutants. After 60 h, the cells were collected and the cell pellets were washed once by cold PBS. Biotinylation was performed through addition of 600 μL 0.8 mg ml^{-1} sulfo-NHS-SS-biotin (Glpbio) and incubation at 4 °C for 30 min with gentle rotation. After this, cells were washed once with 1 mL cold PBS containing 100 mM glycine and incubated for 10 min at 4 °C in the same buffer. The cells were then lysed with 900 μL lysis buffer (20 mM Tris pH 8.0, 150 mM NaCl, 1% (w/v) DDM, 0.2% (w/v) CHS, 0.8 μM aprotinin, 2 μg/ml leupeptin, 2 mM pepstatin A and 1 mM phenylmethylsulfonyl fluoride) for 1 h at 4 °C. Insoluble debris was removed by centrifugation at 40,000g for 20 min and the supernatants were incubated with 50 μL of Strep-Tactin resin for 1 h at 4 °C. The beads were then centrifuged at 500× g for 1 min, washed for twice with ice-cold wash buffer (20 mM Tris pH 8.0, 150 mM NaCl, 0.01% (w/v) LMNG), and incubated with 100 μL of 5× Loading buffer containing 5% β-ME at room temperature for 30 min. Samples were separated using SDS-PAGE

followed by western blot analysis. The NC membranes were preincubated with a blocking buffer (20 mM Tris-HCl, pH 8.0, 150 mM NaCl and 5% milk powder) for 1 h at room temperature, followed by incubation with primary antibodies for 2 h at 4 °C. Biotinylated CRT protein was detected using anti-Flag antibody (Bioworld, 1:10,000 dilution). Anti-Na^+/K^+-ATPase antibody (Abcam, 1:10,000 dilution) was used as a cell membrane marker. After the incubation, the NC membranes were washed 4 times with TBST buffer (20 mM Tris-HCl, pH 8.0, 150 mM NaCl, and 0.1% Tween 20). Then the membranes were respectively incubated with secondary antibodies (800 cw goat anti-mouse/rabbit) for 1 h at room temperature. The membranes were then washed 4 times with TBST buffer. Images were captured using Odyssey CLx.

MD simulation

The CRT structures were acquired through Cryo-EM and the missing loop between H187 and Q202 was constructed using AlphaFold2[7]. At the same time, the pKa values of all the acidic residues were calculated by propKa3[8]. Herein, three residues, namely E109, E379, and D458, were founded to be protonated because their pKa values were higher than the experimental pH value 8. With the help of the CHARMM-GUI web server[9,10], two different CRT protein-ligand systems were setup with the creatine and RGX. Both these two ligands were parameterized by CGenFF with the CHARMM general forcefield[11]. The membrane bilayer was constructed using the POPC lipids and the position of the protein within the membrane bilayer was determined by PPM web server[12]. A total of 219 POPC lipids were added in the system. In addition, about 25 thousand TIP3P[13] water molecules were added around both sides of the membrane bilayer, and the ion (NaCl) concentration was set to 0.15 M. The behaviors of the lipids were described by CHARMM all-atom additive forcefield[14], and the protein was described by the CHARMM36m forcefield[15]. Finally, the AMBER simulation input files were generated for further molecular dynamics (MD) simulations[16,17].

A multi-step process of the traditional MD simulations was implemented to relax the whole system. Initially, a five-thousand-step energy minimization was executed.

During this process, the restraints were applied to the protein and ligand atoms restraints at a magnitude of 10 kcal/mol/Å^2, while the lipids atoms were restrained with a value of 2.5 kcal/mol/Å^2. Simultaneously, the key dihedral angles of the lipid molecules were restrained as well. Subsequently, a 150 ps heating process was carried out to raise the system temperature to 300 K, and another five-step equilibrium process was run to equilibrate the system density under 1 atm pressure. During these five equilibrium steps, the restraints imposed on the system were gradually reduced to zero. Finally, 300 ns MD simulations were carried out under the NPT ensemble. The Langevin thermostat algorithm with a collision frequency of 1.0 ps^{-1} was employed to control the system temperature, and the Monte Carlo barostat was used to control the semi-isotropic pressure[18]. The cutoff value of the nonbonding interaction was to set to 12 Å with the 10 Å switching-function value for van Der Waals interaction. The particle-mesh Ewald (PME) method was chosen to described the long-range summation of the electrostatic interaction. In addition, bonds involving hydrogen were constrained by the SHAKE algorithm[19]. Each of the system was run at least 3 replicates. All the simulations were done with AMBER24[20].

Supplementary References

1. Punjani, A., Rubinstein, J. L., Fleet, D. J. & Brubaker, M. A. cryoSPARC: algorithms for rapid unsupervised cryo-EM structure determination. *Nat Methods* **14**, 290-296 (2017).
2. Goddard, T. D. *et al.* UCSF ChimeraX: Meeting modern challenges in visualization and analysis. *Protein Sci* **27**, 14-25 (2018).
3. Emsley, P. & Cowtan, K. Coot: model-building tools for molecular graphics. *Acta Crystallogr D Biol Crystallogr* **60**, 2126-2132 (2004).
4. Afonine, P. V. *et al.* Real-space refinement in PHENIX for cryo-EM and crystallography. *Acta Crystallogr D Struct Biol* **74**, 531-544 (2018).
5. Chen, V. B. *et al.* MolProbity: all-atom structure validation for macromolecular crystallography. *Acta Crystallogr D Biol Crystallogr* **66**, 12-21 (2010).
6. Goehring, A. *et al.* Screening and large-scale expression of membrane proteins in mammalian cells for structural studies. *Nat Protoc* **9**, 2574-2585 (2014).
7. Jumper, J. *et al.* Highly accurate protein structure prediction with AlphaFold. *Nature* **596**, 583-589 (2021). https://doi.org:10.1038/s41586-021-03819-2
8. Olsson, M. H. M., Sondergaard, C. R., Rostkowski, M. & Jensen, J. H. PROPKA3: Consistent Treatment of Internal and Surface Residues in Empirical Predictions. *J Chem Theory Comput* **7**, 525-537 (2011). https://doi.org:10.1021/ct100578z
9. Jo, S., Klauda, J. B. & Im, W. CHARMM-GUI Membrane Builder for Mixed Bilayers and Its Application to Yeast Membranes. *Biophys J* **96**, 41a-41a (2009).
10. Park, S., Choi, Y. K., Kim, S., Lee, J. & Im, W. CHARMM-GUI Membrane Builder for Lipid Nanoparticles with Ionizable Cationic Lipids and PEGylated Lipids. *J Chem Inf Model* **61**, 5192-5202 (2021). https://doi.org:10.1021/acs.jcim.1c00770
11. Vanommeslaeghe, K. *et al.* CHARMM General Force Field: A Force Field for Drug-Like Molecules Compatible with the CHARMM All-Atom Additive

Biological Force Fields. *J Comput Chem* **31**, 671-690 (2010). https://doi.org:10.1002/jcc.21367

12 Lomize, M. A., Pogozheva, I. D., Joo, H., Mosberg, H. I. & Lomize, A. L. OPM database and PPM web server: resources for positioning of proteins in membranes. *Nucleic Acids Res* **40**, D370-D376 (2012). https://doi.org:10.1093/nar/gkr703

13 Mark, P. & Nilsson, L. Structure and dynamics of the TIP3P, SPC, and SPC/E water models at 298 K. *J Phys Chem B* **105**, 24a-24a (2001).

14 Venable, R. M. *et al.* CHARMM All-Atom Additive Force Field for Sphingomyelin: Elucidation of Hydrogen Bonding and of Positive Curvature. *Biophys J* **107**, 134-145 (2014). https://doi.org:10.1016/j.bpj.2014.05.034

15 Huang, J. *et al.* CHARMM36m: an improved force field for folded and intrinsically disordered proteins. *Nat Methods* **14**, 71-73 (2017). https://doi.org:10.1038/Nmeth.4067

16 Lee, J. *et al.* CHARMM-GUI supports the Amber force fields. *J Chem Phys* **153** (2020).

17 Lee, J. *et al.* CHARMM-GUI Input Generator for NAMD, GROMACS, AMBER, OpenMM, and CHARMM/OpenMM Simulations Using the CHARMM36 Additive Force Field. *J Chem Theory Comput* **12**, 405-413 (2016). https://doi.org:10.1021/acs.jctc.5b00935

18 Bernetti, M. & Bussi, G. Pressure control using stochastic cell rescaling. *J Chem Phys* **153**, 114107 (2020). https://doi.org:10.1063/5.0020514

19 Macuglia, D. SHAKE and the exact constraint satisfaction of the dynamics of semi-rigid molecules in Cartesian coordinates, 1973-1977. *Arch Hist Exact Sci* **77**, 345-371 (2023). https://doi.org:10.1007/s00407-023-00306-0

20 AMBER 2024 (University of California, San Francisco, 2024).

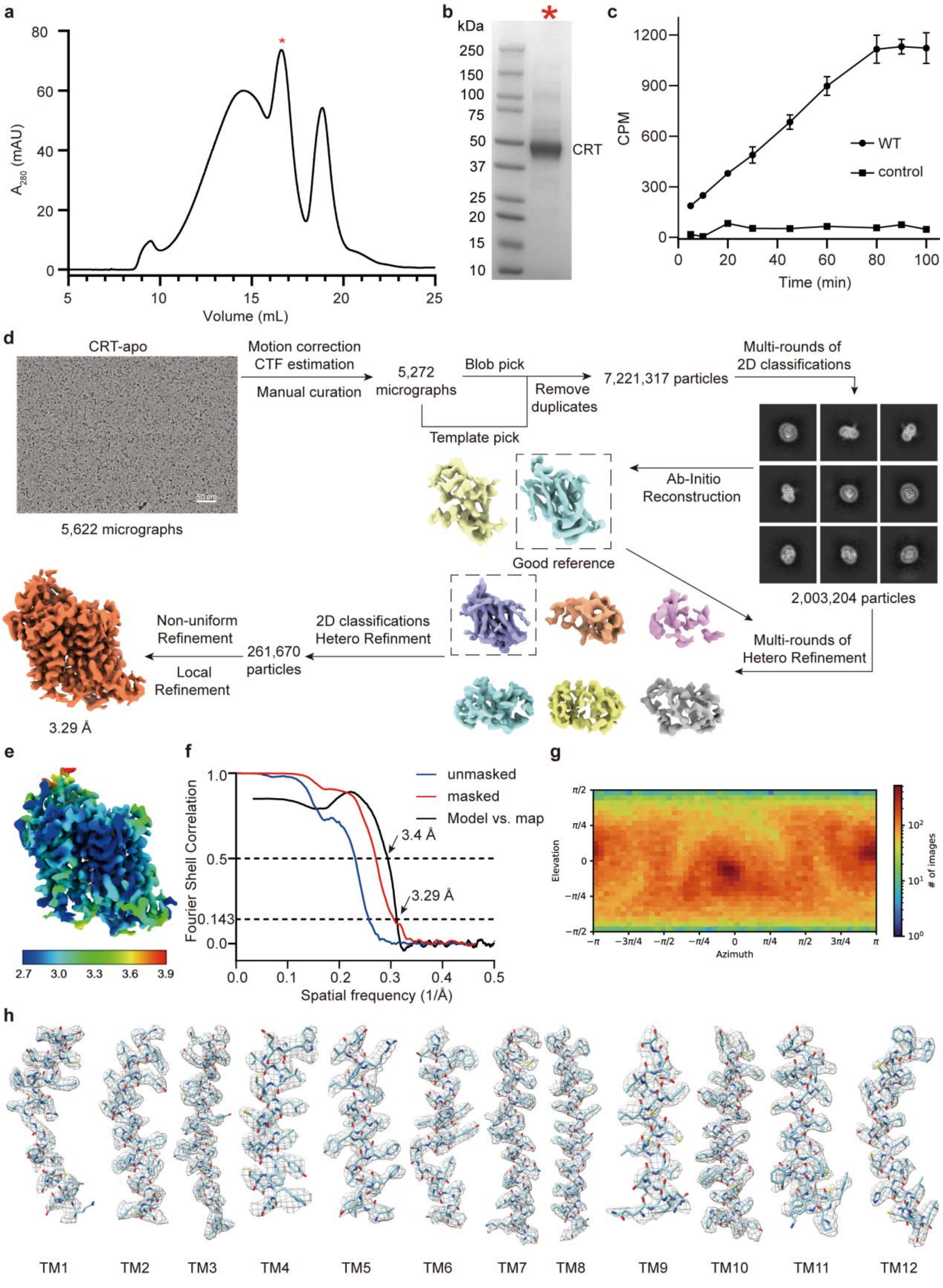

Supplementary Fig. S1 Protein purification and Cryo-EM reconstruction of CRT-apo.

a. Size-exclusion chromatography of purified human CRT protein sample. The fractions were pooled for cryo-EM sample preparation.

b. SDS-PAGE gel of purified human CRT.

c. Time-dependent uptake of [^{14}C]creatine by CRT. Uptake mediated by CRT-WT. Cells without transfection were used as blank control. Data are represented as mean ±SEM; n = 3 biologically independent experiments.

d. Data processing flowchart of CRT-apo in the inward-open conformation. A representative micrograph was shown with the white bar equaling 50 nm. Further details can be found in Methods. The contour level of final map is 0.35.

e. Local resolution map of CRT-apo, colored according to the estimated value ranging from 2.7 to 3.9 Å.

f. Gold standard Fourier Shell Correlations (FSC) curves of unmasked map (blue), masked map (red) and model versus map (black), marked with resolutions corresponding to FSC = 0.5 and 0.143.

g. Angular sampling of the final reconstruction, generated from cryoSPARC.

h. The cryo-EM densities of TM1-12 in CRT-apo, contoured at 0.35.

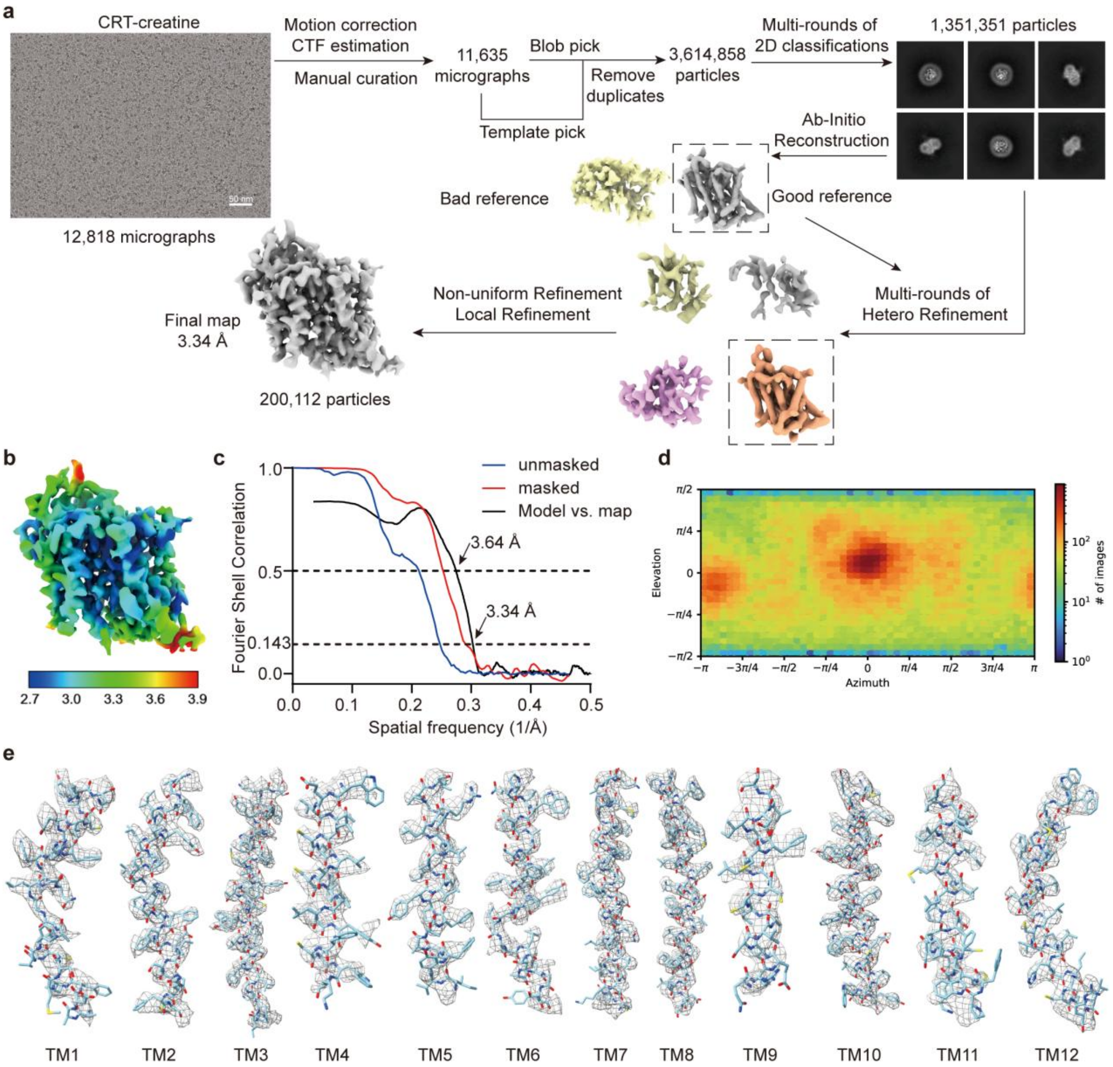

Supplementary Fig. S2 Cryo-EM reconstruction of CRT-creatine.

a. Data processing flowchart of CRT-creatine in the inward-occluded conformation. A representative micrograph was shown with the white bar equaling 50 nm. Further details can be found in Methods. The contour level of final map is 0.35.

b. Local resolution map of CRT- creatine, colored according to the estimated value ranging from 2.7 to 3.9 Å.

c. Gold standard Fourier Shell Correlations (FSC) curves of unmasked map (blue), masked map (red) and model versus map (black), marked with resolutions corresponding to FSC = 0.5 and 0.143.

d. Angular sampling of the final reconstruction, generated from cryoSPARC.

e. The cryo-EM densities of TM1-12 in CRT-creatine, contoured at 0.35.

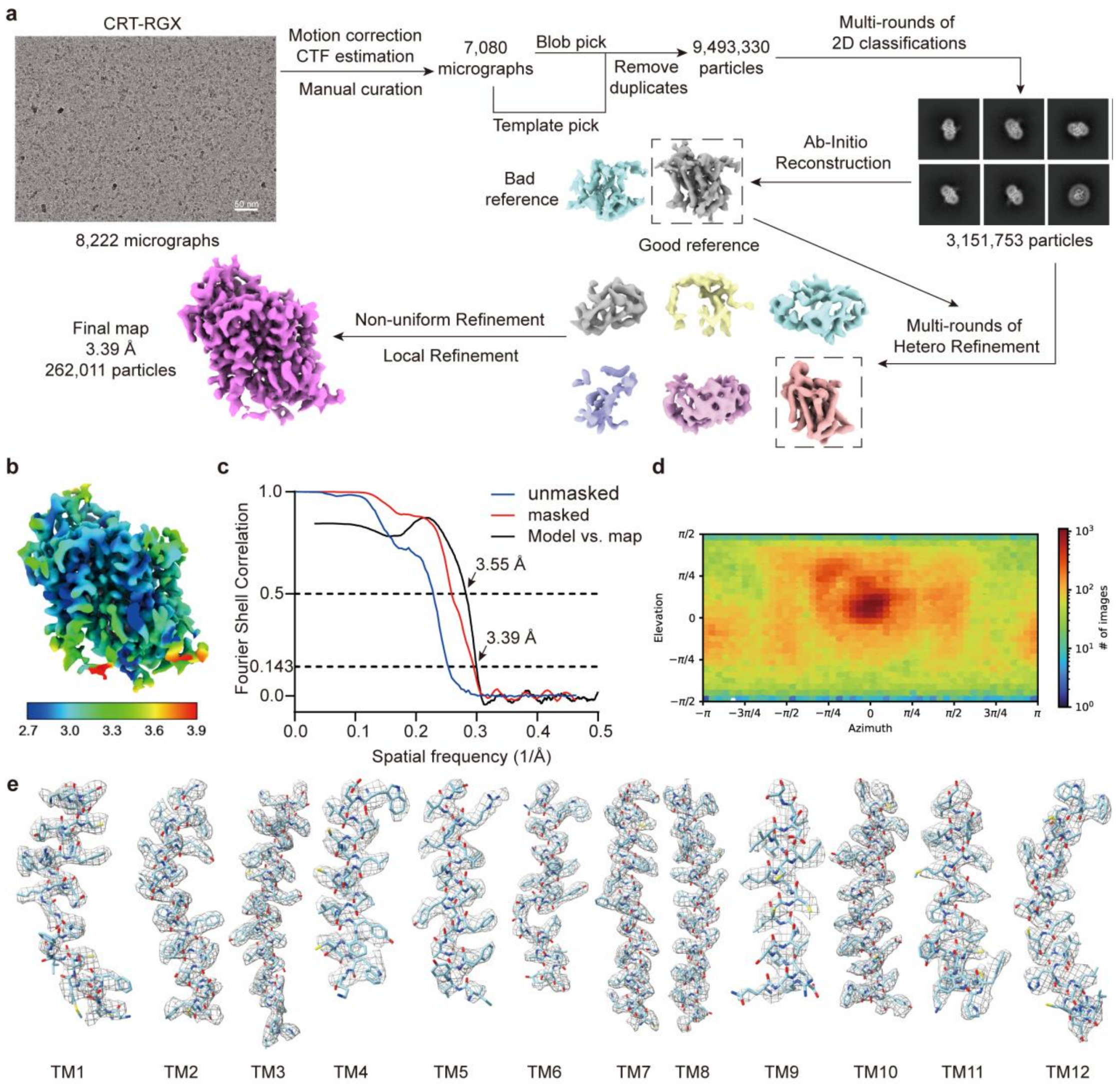

Supplementary Fig. S3 Cryo-EM reconstruction of CRT-RGX-202.

a. Data processing flowchart of CRT-RGX-202 in the inward-occluded conformation. A representative micrograph was shown with the white bar equaling 50 nm. Further details can be found in Methods. The contour level of final map is 0.35.

b. Local resolution map of CRT- RGX, colored according to the estimated value ranging from 2.7 to 3.9 Å.

c. Gold standard Fourier Shell Correlations (FSC) curves of unmasked map (blue), masked map (red) and model versus map (black), marked with resolutions corresponding to FSC = 0.5 and 0.143.

d. Angular sampling of the final reconstruction, generated from cryoSPARC.

e. The cryo-EM density and atomic model of TM1-12 of CRT-RGX, contoured at 0.35.

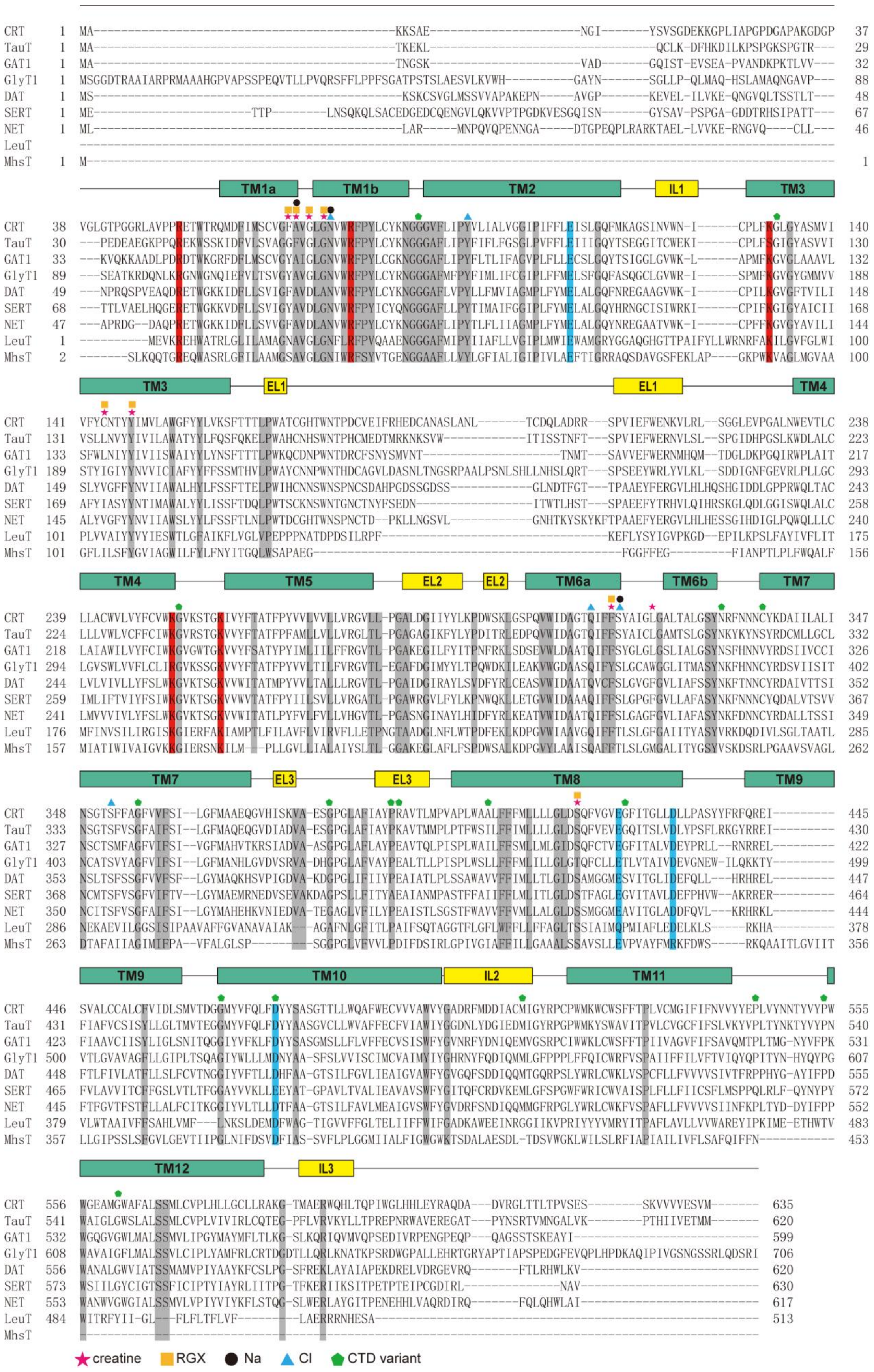
CRT
TauT
GAT1
GlyT1
DAT
SERT
NET
LeuT
MhsT
TM1a
TM1b
TM2
IL1
TM3
EL1
TM4
TM5
EL2
TM6a
TM6b
TM7
EL3
TM8
TM9
TM10
IL2
TM11
TM12
IL3
creatine
RGX
Na
Cl
CTD variant

Supplementary Fig. S4 Sequence alignment of CRT, TauT, GAT1, GlyT1, DAT, SERT, NET, LeuT, and MhsT.

Secondary structural elements of CRT are indicated above the sequence alignment. Highly conserved amino acids are shaded red, blue, and gray for positively charged, negatively charged and others, respectively. Residues involved in the interactions with creatine, RGX-202, Na^{+} ion, Cl^{-} ion, and CTD-related variants are labeled by different symbols above residues. The UniProt (https://www.uniprot.org) IDs for the aligned proteins are: CRT(P48029), TauT (P31641), GAT1(P30531), GlyT1(P48067), DAT(Q01959), SERT(P31645), NET(P23975), LeuT(O67854) and MhsT(Q9KDT3).

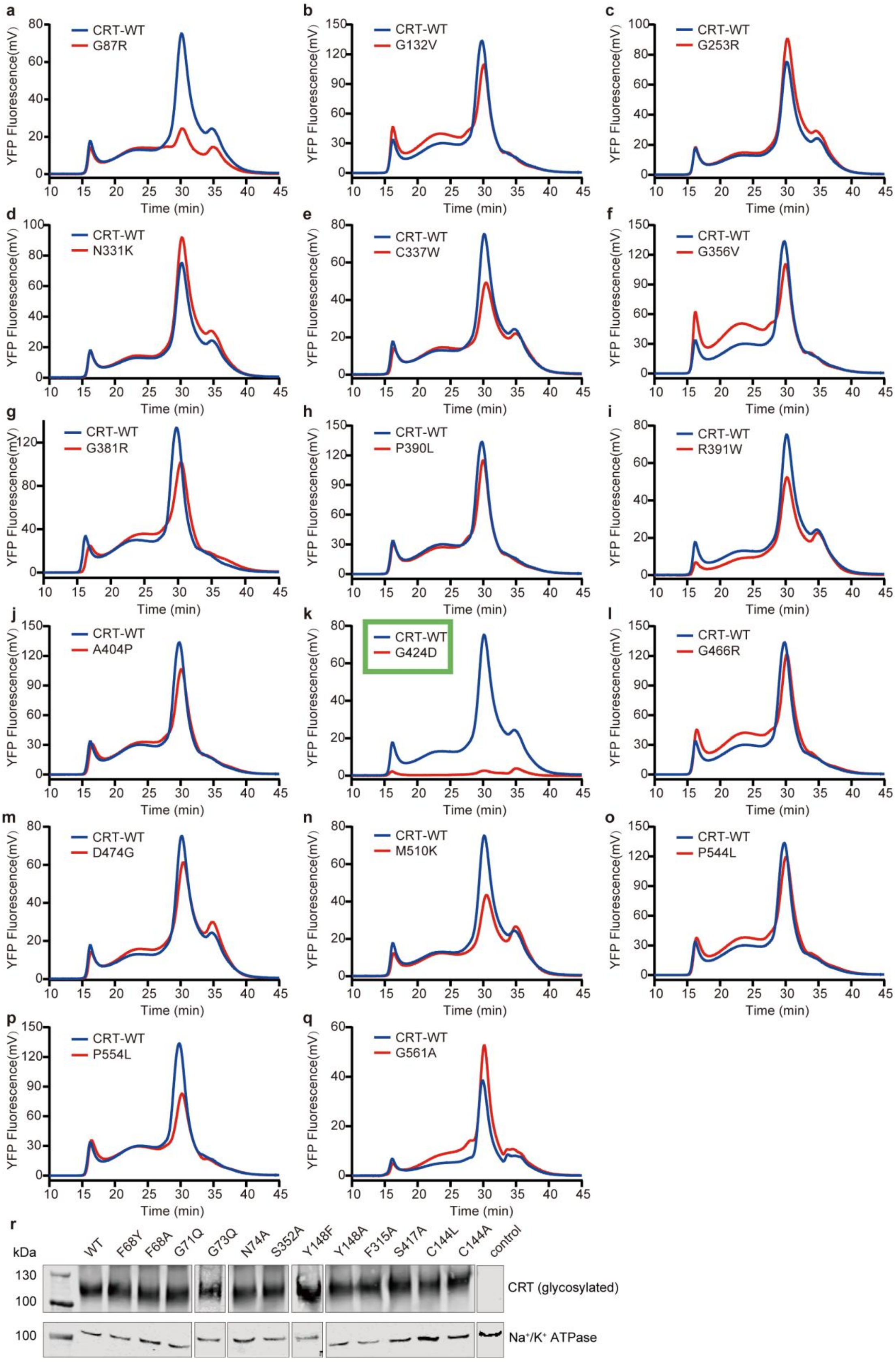

Supplementary Fig. S5 Fluorescence size exclusion chromatography profiles of wild type CRT (blue color) and its CTD-related variants (red color) (a-q) and

surface expression of CRT WT and mutants (r). Surface expression levels are shown in western blotting for WT, mutants and control. Control indicates empty cell control. Biotinylated Na^+/K^+ ATPase on the cell surface was used as the loading control.

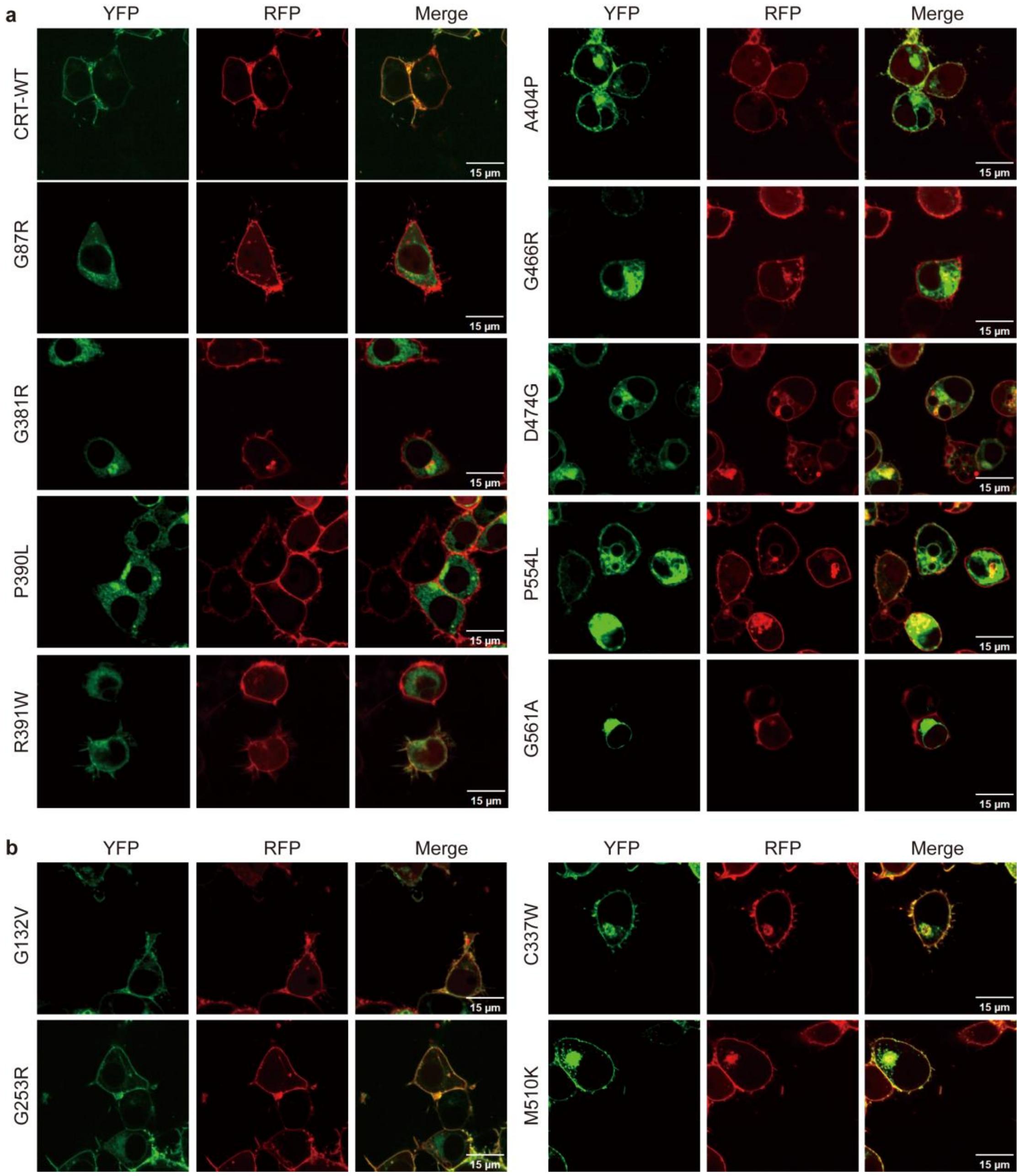

Supplementary Fig. S6 Subcellular localization of CTD-related variants in CRT.
CRT-WT and CTD-related CRT variants were shown in green and neuromodulin (plasma membrane marker) were shown in red. Colocalization of the two signals is shown in the merged images. Colocalization of wild-type CRT or its variants with the plasma membrane was quantified using Pearson's correlation coefficient, where a value of 1 indicates perfect colocalization and a value of -1 denotes an inverse distribution.

a. Mutations located on the extracellular side of CRT were shown. Pearson's coefficient values for co-localization of plasma membrane and the YFP-tagged CRT

were: 0.8126 (CRT-WT), -0.0097 (G87R), -0.1003 (G381R), -0.0297 (P390L), 0.1712 (R391W), 0.5195 (A404P), -0.0239 (G466R), 0.5684 (D474G), 0.3488 (P554L), 0.0731 (G561A).

b. Mutations located on the intracellular side of CRT were shown. Pearson's coefficient values were: 0.5832 (G132V), 0.8377 (G253R), 0.7187 (C337W), 0.6624 (M510K).

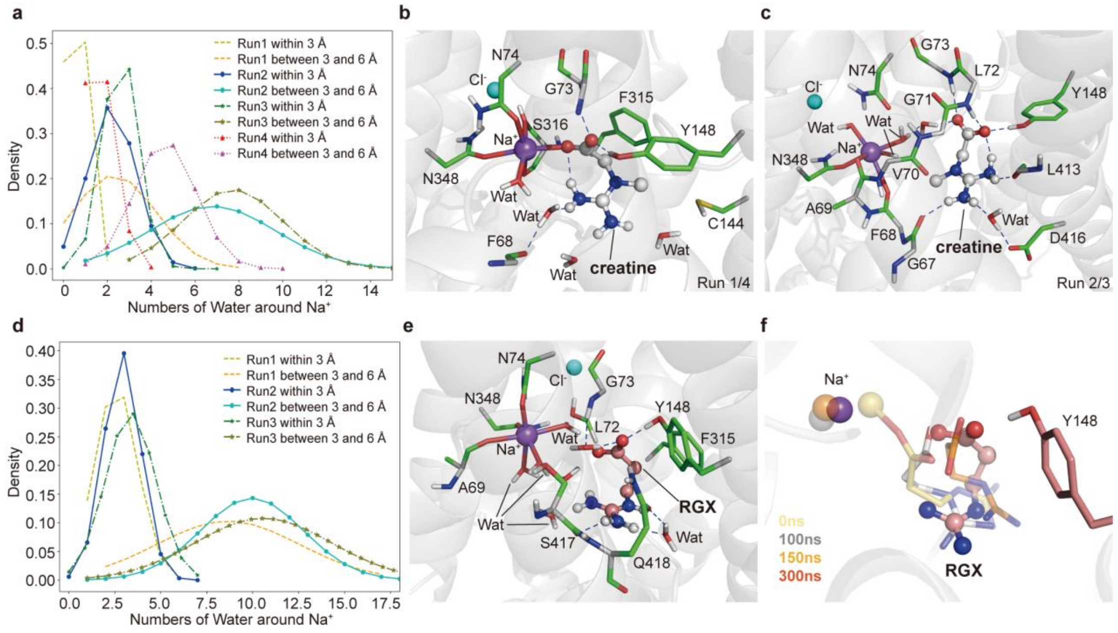

Supplementary Fig. S7 The MD simulations of the creatine and RGX binding to CRT.

a. The coordination of Na^+ ions with water molecules in four simulations of the creatine binding to CRT. At most 2 water molecules are able to reach the Na^+ ion first coordination shell (within 3Å of Na^+ ion) and about 6 water molecules are in the second coordination shell (between 3Å and 6Å of Na^+ ion) in Run1 and Run4. The Na^+ ions directly bind to at least 3 water molecules, and the second coordination shell is saturated by about 8 to 10 water molecules in Run2 and Run3. **b.** The binding mode of the creatine in Run1 and Run4. **c.** The binding mode of the creatine in Run2 and Run3. **d.** The coordination of Na^+ ions with water molecules in three simulations of the RGX binding to CRT. In Run1-3, the Na^+ ions coordinate to about 3 water molecules in the first coordination shell, and the second coordination shell is filled by about 10 water molecules. **e.** The binding mode of the RGX. **f.** The key frames in one of the RGX simulations. RGX in different simulation time (0, 100, 150, 300 ns) are colored in yellow, grey, orange and violet, respectively. Na^+ ions in different simulation time (0, 100, 150, 300 ns) are displayed as yellow, grey, orange and purple spheres, respectively.

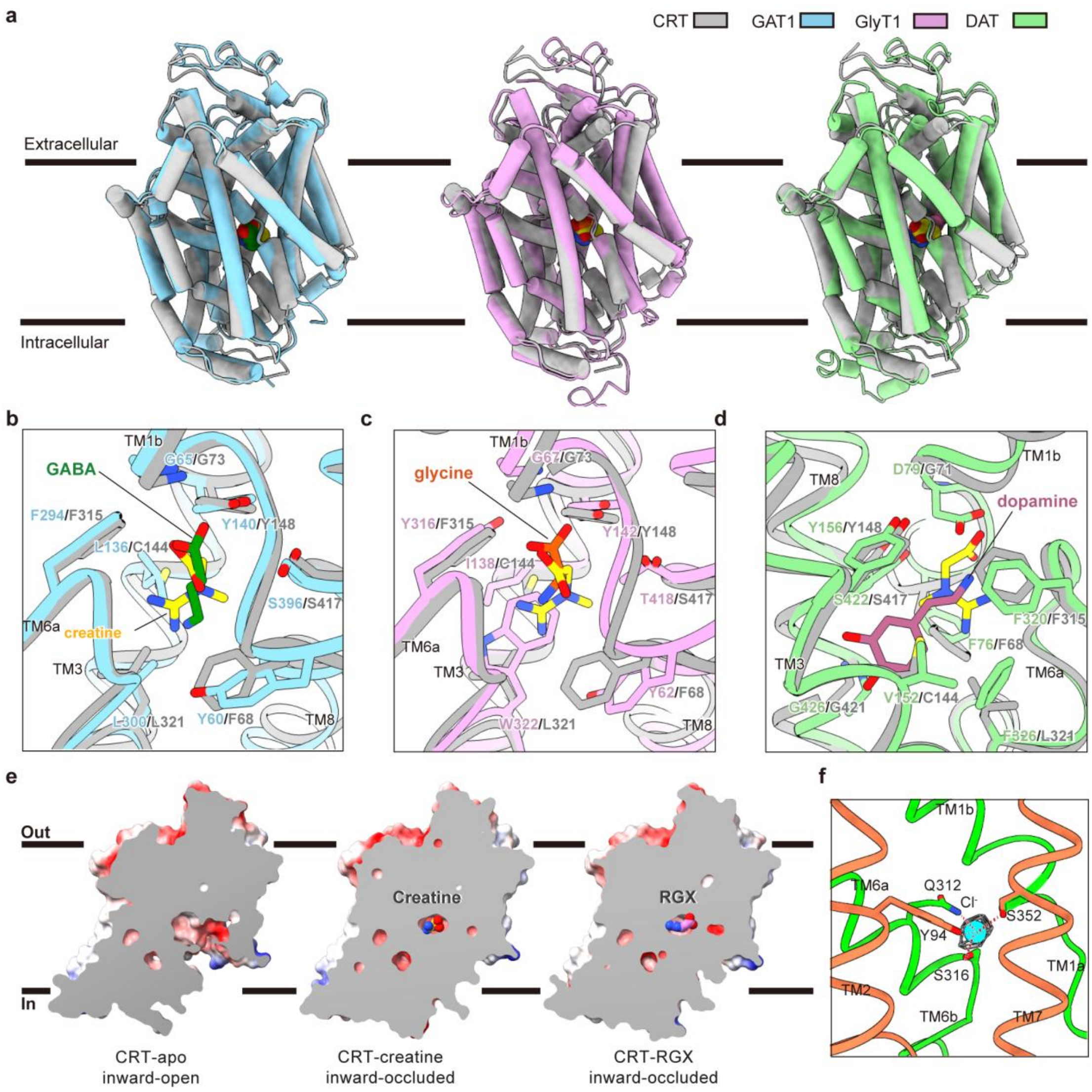

Supplementary Fig. S8 Structural comparison of CRT-creatine to substrate-bound NSS members, cross section of electrostatic surface of CRT and the coordinations of Cl^- ion in the central binding site of CRT-apo.

a. Overall structural comparisons of CRT with GAT1, DAT and GlyT1, respectively. The creatine-bound CRT, GABA-bound GAT1 (PDB code: 7Y7W), glycine-bound GlyT1 (PDB code: 8WFI) and dopamine-bound DAT (PDB code: 8Y2D) are represented by grey, blue, violet and green cartoon, respectively.

b-d. Superimposing the binding pocket of CRT with those of GAT1-GABA (**b**), GlyT1-glycine (**c**), and DAT-dopamine (**d**), The substrates creatine, GABA, glycine and dopamine are colored in yellow, green, coral and purple, respectively.

e. Cross section of electrostatic surface of CRT. Substrates are showed as ball sticks

and labeled.

f. The coordinations of Cl^- ion in the central binding site of CRT-apo. Residues that participate in the interaction are shown as sticks and labeled. The contour level is 0.35.

Supplementary Table S1 Statistics of 3D reconstructions and model refinement.

**Protein**	CRT-apo	CRT-creatine	CRT-RGX-202
**Data collection and processing**			
Magnification	81K	81K	81K
Voltage （kV）	300	300	300
Electron exposure ($e^{-}/Å^2$)	50	50	50
Defocus range (μm)	-1.0 ~ -2.0	-1.0 ~ -2.0	-1.0 ~ -2.0
Magnified pixel size (Å)	1.055	1.055	1.055
Micrographs	5,622	12,818	8,222
Initial particle images (no.)	2,003,204	3,614,858	3,151,753
Final particle images (no.)	261,670	288,078	262,011
Symmetry	C1	C1	C1
Map Resolution (Å)	3.29	3.40	3.39
Sharpening B-factor ($Å^2$)	151.9	152.1	156.8
**Model statistics**			
Map CC	0.79	0.77	0.79
Number of atoms	4261	4234	4236
**R.m.s. deviations**			
Bond length (Å)	0.004	0.004	0.005
Bond angle (°)	0.530	0.656	0.600
**Ramachandran plot**			
Favored (%)	95.66	95.45	96.21
Allowed (%)	4.34	4.55	3.79
Disallowed (%)	0.00	0.00	0.00
Rotamers Outlier (%)	0.00	0.00	0.00
MolProbity Score	1.74	1.90	1.80
